# Different Long-Term Duration of Seroprotection against *Neisseria meningitidis* in Adolescents and Middle-Aged Adults after a Single Meningococcal ACWY Conjugate Vaccination in The Netherlands

**DOI:** 10.3390/vaccines8040624

**Published:** 2020-10-25

**Authors:** Milou Ohm, Debbie M. van Rooijen, Axel A. Bonačić Marinović, Mariëtte B. van Ravenhorst, Marieke van der Heiden, Anne-Marie Buisman, Elisabeth A.M. Sanders, Guy A.M. Berbers

**Affiliations:** 1 Centre for Infectious Disease Control (Cib), National Institute of Public Health and the Environment (RIVM), 3720 BA Bilthoven, The Netherlands; debbie.van.rooijen@rivm.nl (D.M.v.R.); axel.bonacic.marinovic@rivm.nl (A.A.B.M.); annemarie.buisman@rivm.nl (A.-M.B.); lieke.sanders@rivm.nl (E.A.M.S.); guy.berbers@rivm.nl (G.A.M.B.); 2 Department of Pediatrics, Amsterdam UMC, 1007 MB Amsterdam, The Netherlands; mvravenh@gmail.com; 3 Department of Molecular Biosciences, The Wenner-Gren Institute, Stockholm University, 114 18 Stockholm, Sweden; mariekevdheiden@gmail.com; 4 Department of Pediatric Immunology and Infectious Diseases, Wilhelmina Children’s Hospital, University Medical Center, 3584 CX Utrecht, The Netherlands

**Keywords:** *Neisseria meningitidis*, adolescents, middle-aged adults, quadrivalent meningococcal vaccine, serum bactericidal antibody assay, long-term protection

## Abstract

*Neisseria meningitidis* is often asymptomatically carried in the nasopharynx but may cause invasive meningococcal disease, leading to morbidity and mortality. Meningococcal conjugate vaccinations induce functional protective antibodies against capsular antigens, but seroprotection wanes over time. We measured functional antibody titers five years after administration of a single dose of the meningococcal ACWY-polysaccharide-specific tetanus toxoid-conjugated (MenACWY-TT) vaccine in adolescents and middle-aged adults in the Netherlands, using the serum bactericidal antibody with baby rabbit complement (rSBA) assay. Protection was defined as rSBA titer ≥8. The meningococcal ACWY-specific serum IgG concentrations were measured with a multiplex immunoassay. Duration of protection was estimated by a bi-exponential decay model. Sufficient protection for MenC, MenW, and MenY was achieved in 94–96% of the adolescents five years postvaccination, but, in middle-aged adults, only in 32% for MenC, 65% for MenW and 71% for MenY. Median duration of protection for MenCWY was 4, 14, and 21 years, respectively, in middle-aged adults, while, in adolescents, it was 32, 98, and 33 years. Our findings suggest that adolescents, primed in early childhood with MenC conjugate vaccination, remain sufficiently protected after a single dose of MenACWY-TT vaccine. Middle-aged adults without priming vaccination show fast waning of antibodies, particularly MenC, for which protection is lost after four years.

## 1. Introduction

As a commensal bacterium, *Neisseria meningitidis* resides in the nasopharynx in humans mostly without clinical symptoms. However, sometimes encapsulated serogroups may invade the bloodstream of the human host, and cause invasive meningococcal disease (IMD) [1,2]. IMD has both severe acute and life-long consequences and is a major cause of mortality [3]. Antibiotics are the main therapy, together with supportive therapy, but may work too late against this rapidly progressive disease. To prevent meningococcal disease, vaccination with meningococcal conjugate vaccines induces the production of protective antibodies against the polysaccharide capsule of the meningococcal bacterium [4]. Although a vaccine also induces a cellular memory response, the response might be too slow to provide protection against *Neisseria meningitidis.* A memory response can take up to five days, while an invasive disease can manifest itself within hours after encountering the pathogen [5,6]. For protection, it is therefore necessary to maintain sufficient levels of circulating anticapsular antibodies that directly interact with the complement system to prevent invasive disease by bacterial killing [7,8,9,10].

A steep rise in meningococcal C (MenC) disease incidence around 2000 in the Netherlands led to the introduction of a single MenC tetanus toxoid conjugate (MenC-TT) vaccination at 14 months of age in the national immunization program (NIP) in 2002 [11]. In addition, a MenC-TT vaccination was offered to all children aged 1–18 years as part of a catch-up mass-campaign to eradicate MenC circulation. A single-dose schedule at 14 months of age did not provide sufficient protection on the long-term [12] and the timing of an adolescent booster-dose in the NIP was investigated [13,14]. In 2015/2016, a MenW epidemic in the Netherlands emerged, which led to the introduction of meningococcal A, C, W, and Y conjugated to tetanus toxoid (MenACWY-TT) vaccine for toddlers at age 14 months with a booster vaccination at the age of 14 years. At the same time, a catch-up campaign with a single dose of the MenACWY-TT vaccine was conducted in all 14 to 18-year-olds. Although this campaign is assumed to include the main carriers of MenW [15] and, in this way, might eventually lead to a benefit for unvaccinated individuals through herd protection; this may take time [16]. Disease cases in the MenW epidemic occurred not only in the very young children and in adolescents, but also in adults and in the elderly [17]. To protect older age groups directly against IMD, vaccination of other age categories might be required [18]. In former studies, MenACWY-TT vaccination has shown to elicit a good immune response in both children and adults [18,19,20,21,22,23,24]. However, we have previously shown that the functional antibody titers in middle-aged adults were lower compared to adolescents already at one month after vaccination [25].

To optimize vaccination strategies, knowledge about the duration of protection after a single MenACWY-TT vaccination is essential. However, long-term persistence of functional antibodies induced by a MenACWY-TT vaccination has been scarcely investigated, especially in older adults [26,27]. The level of vaccine-induced protection and potentially also the duration of protection seems to vary among different age groups [22,28]. The aim of the current study was to determine duration of protection after a single MenACWY-TT vaccination in adolescents who were once primed with a single MenC-TT vaccination at preschool age and middle-aged adults who were naïve to meningococcal vaccination. We assessed both functional antibody titers and concentrations of IgG antibodies using a five-year postvaccination serum sample and estimated vaccine-induced duration of protection in years using a multilevel bi-exponential decay model. Furthermore, meningococcal type-specific IgG concentrations were compared with rSBA titers of the corresponding serogroup to gain insight into the difference between quantity and functionality of persisting antibodies.

## 2. Materials and Methods

### 2.1. Study Design and Participants

This is a five-year follow-up study of two phase-IV trials conducted in a single center in the Netherlands. In these trials, a primary MenACWY-TT vaccination was administered to 225 healthy adolescents who were all once primed with a MenC-TT vaccine (NeisVac-C) at an age between 14 months and 3 years, and to 204 healthy middle-aged adults who were naïve to meningococcal vaccination. Detailed information on recruitment, study design, in-and exclusion criteria and clinical procedures are previously described [18,29]. In short, in the adolescent trial (EudraCT number: 2013-001823-38, Dutch Trial Register: NL4286), healthy 10-, 12-, and 15-year-olds were recruited in the surrounding area of Utrecht, The Netherlands. All participants received a single dose of the MenACWY-TT vaccine in the spring of 2014. Venous blood samples were collected before, 1 month, and 1 year after the study vaccination. In the middle-aged adult trial (EudraCT number: 2014-000967-42, Dutch Trial Register: NL4518), healthy 50- to 65-year-olds were recruited in the municipality of Amersfoort, the Netherlands. All participants received a single dose of the MenACWY-TT vaccine in the autumn of 2014. Venous blood samples were collected before, 7 days, 1 month, and 1 year after the study vaccination.

In the follow-up studies, all participants that completed the former trial and gave permission to approach them in the future were asked to donate a venous blood sample that was collected 5 years (±3 months) after vaccination. Receiving an additional MenACWY vaccination after the 1-year timepoint was now added to the exclusion criteria. Participants that failed to build up an immune response after the MenACWY-TT vaccination during the adolescent study were excluded because they were offered an extra vaccination.

These studies were designed and conducted in accordance with the Good Clinical Practice guidelines established by the International Conference on Harmonization and with the Declaration of Helsinki. Ethical approval was obtained from the local ethics committee Medical research Ethics Committees United (MEC-U) for both follow-up studies. The middle-aged adult study was approved as an amendment. Since the adolescent study was already officially terminated in the national study register, this follow-up study was registered separately at the Dutch Trial Register (NL7735). Written informed consent was obtained from all participants and from both parents or guardians when a subject was aged <16 years at enrolment.

### 2.2. Serological Analysis

The functional antibodies were assessed by performing a serum bactericidal antibody with baby rabbit complement (rSBA) assay (Pelfreez, Rogers, Arkansas, U.S.A, lot 22841) and MenC strain C11 [30], MenW strain MP01240070, and MenY strain S-1975 as target strains. The serum bactericidal titer was defined as the dilution of the test serum that yielded ≥50% killing after 60 min incubation with a titer of ≥8 as correlate of protection [31,32,33]. Functional antibody titers were also analyzed using the more conservative threshold of ≥128 [31,32]. For statistical purposes, rSBA titers below the cut-off of the assay (<4) were given a value of 2. Since no data were available from the former study in middle-aged adults about MenA titers, the MenA rSBA assay was not performed in this follow-up study. MenA-, MenC-, MenW-, and MenY-PS specific serum IgG concentrations were measured using the fluorescent-bead-based multiplex immunoassay (MIA) as previously described [34,35,36], with minor modification of using a protein-free buffer (Surmodics, Eden Prairie, MN, U.S.A.) since 2019. The lower limit of quantitation was assigned at 0.01 µg/mL for all four serogroups [34,35,36]. A previously suggested, arbitrary cut-off of ≥2 µg/mL for total serum IgG was used for analyses [37,38,39,40,41].

### 2.3. Mathematical Model

A multilevel bi-exponential decay model was used to estimate the long-term protection in terms of functional antibody persistence [42,43,44,45]. This model describes the rSBA titer decay with the following equation:

(1) $$Y\left(t\right)=Y_{1}\left(\frac{e^{-\nu_{1}\left(t-t_{1}\right)}+f_{y}e_{\;}^{-\nu_{2}\left(t-t_{1}\right)}}{1+f_{y}}\right)$$

where *Y*(*t*) is the antibody titer as function of time (*t*) after reaching its peak concentration at time t_1_. $\nu_{1}$ and $\nu_{2}$ are the rates of the two exponential decay components conforming the bi-exponential model. After the antibody level peaks at time *t*_1_ with value $Y_{1}$, its decay rate is dominated by the faster decay rate $\nu_{1}$. After a while, depending on the value of the factor $f_{y}$, the antibody level decay rate slows down and ends up being dominated by the slower decay rate, $\nu_{2}$. The individual antibody titers of each participant at four timepoints (just before, one month, one year, and five years after vaccination) were used to inform the model under a Bayesian framework. By means of Markov chain Montecarlo simulations, the model parameters were calculated and used to predict expected rSBA titers as a function of time. Four million iterations per simulation were calculated using the software JAGS [46], version 4.3, run under R, version 3.6.3 (https://www.r-project.org/).

### 2.4. Statistical Analysis

All statistical analyses were performed using GraphPad Prism 8 and SPSS Statistics 24. rSBA geometric mean titers (GMTs) and geometric mean concentrations (GMCs) of the meningococcal specific IgG concentrations were calculated with corresponding 95% confidence intervals (CI). Differences between age groups in GMTs and in GMCs at the five-year timepoint, and for the GMTs also at the pre-vaccination timepoint (T0) were determined with the Mann–Whitney test. Differences between age groups in GMTs at the timepoints one month and one year postvaccination were determined with linear regression analyses on natural log-transformed values, adjusting for pre-vaccination values from the former studies. Proportions with 95% CI of participants with a rSBA ≥8 and ≥128 were calculated using the Wilson score interval with continuity correction [47]. Differences in proportions at the five-year timepoint were tested with the Fisher’s exact test. The Spearman correlation coefficient (R) was calculated to compare rSBA titers and IgG concentrations. Statistical tests were 2-sided. A calculated *p*-value below 0.05 was considered statistically significant.

## 3. Results

### 3.1. Study Participants

**Figure 1:**
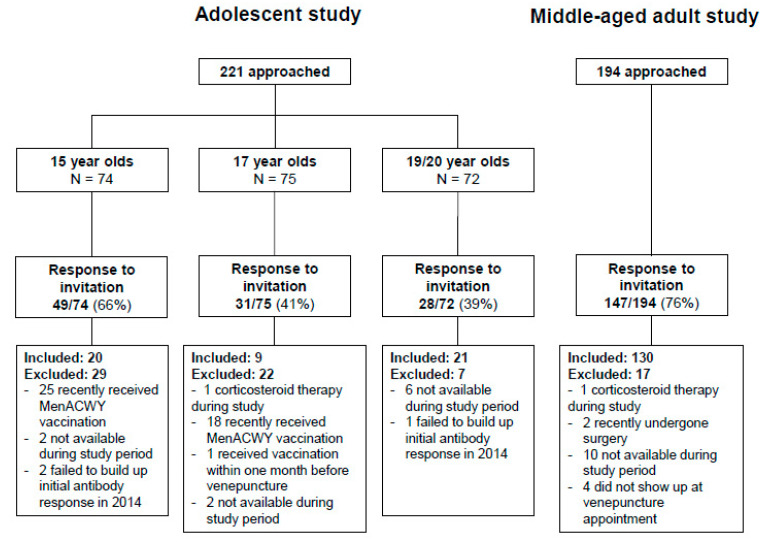
Flow-chart for response to invitation, inclusion and exclusion in the follow-up studies.

Of the 225 participants that received a MenACWY-TT vaccination in the adolescent study [29], 221 were approached with an invitation to participate again. Many former participants who were interested in participating had to be excluded from this study due to a recently received MenACWY-TT vaccination as part of the mass-campaign in the Netherlands; therefore, only 50 could be included in the current follow-up study. Of the 204 participants in the middle-aged adult study [18], 194 were approached with an invitation to participate again and 130 could be included in the follow-up study. In total, 180 participants were enrolled in the follow-up studies (Figure 1).

### 3.2. Persistence of Antibodies after MenACWY-TT Vaccination

**Figure 2:**
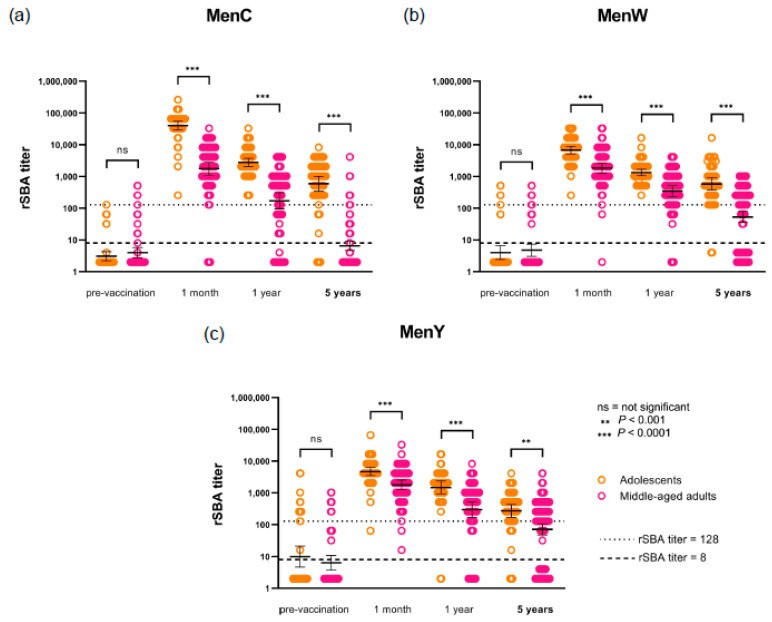
Longitudinal meningococcal serogroup C (**a**), W (**b**) and Y (**c**) specific geometric mean titers (GMTs) of serum bactericidal antibody with baby rabbit complement (rSBA) for two age groups at pre-vaccination, 1 month, 1 year, and 5 years after a meningococcal serogroup A, C, W, Y conjugated to tetanus toxoid (MenACWY-TT) vaccination. Error bars indicate 95% confidence intervals. The orange and pink dots represent the individual measured titers. *p*-values for the pre-vaccination timepoint and five-year timepoint were calculated with Mann–Whitney test. *p*-values for the 1 month and 1 year timepoint were calculated on natural log-transformed values with linear regression, adjusting for pre-vaccination titers.

Five years postvaccination, protective rSBA titers ≥8 were observed in 94% (MenC), 96% (MenW) and 94% (MenY) of the adolescents (Figure 2). Protection against all three serogroups was present in 88% of the participants.

Middle-aged adults showed rSBA titers ≥8 against MenC in 32%, against MenW in 65% and against MenY in 71% (Figure 2 and Table 1) at the five-year timepoint. Only 19% of the participants were still protected against all three serogroups after five years. The meningococcal specific GMTs differed significantly between the age groups not only at 1 month and 1 year but now also at five years after vaccination (Table 1). The proportion of adolescents showing rSBA titers above the more conservative threshold of ≥128 was significantly higher for all serogroups compared with the proportion of protected middle-aged adults (Table 1). A considerable difference was seen for MenC, as 88% of the adolescents showed rSBA titers ≥128 while only 13% of the middle-aged adults showed titers above this more conservative threshold (Table 1).

**Figure 3:**
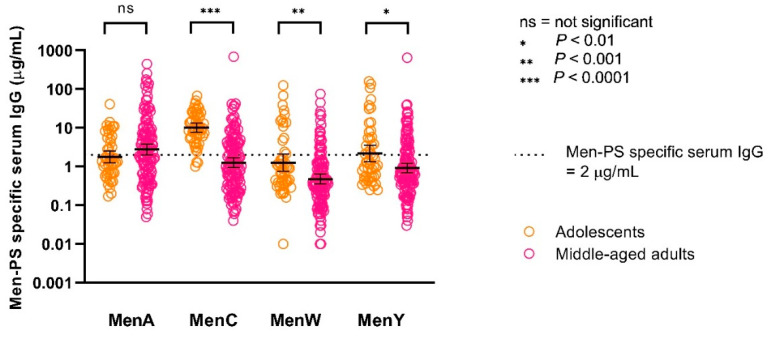
MenA, MenC-, MenW-, and MenY-PS specific serum IgG concentrations five years after a meningococcal serogroup A, C, W, Y conjugated to tetanus toxoid (MenACWY-TT) vaccination. Error bars indicate 95% confidence intervals. The orange and pink dots represent the individual measured concentrations. *p*-values were calculated using the Mann–Whitney test.

The adolescents showed significantly higher MenC-PS, MenW-PS, and MenY-PS specific serum IgG concentrations compared to the middle-aged adults, while, for MenA, no significant difference between the age groups was observed (Table 1 and Figure 3). While the GMTs of the three serogroups in adolescents were comparable or at most 2-fold higher, the GMC of MenC was almost 8-fold and 5-fold higher than the GMC of MenW and MenY, respectively.

### 3.3. Waning of Functional Antibodies and Duration of Protection

**Figure 4:**
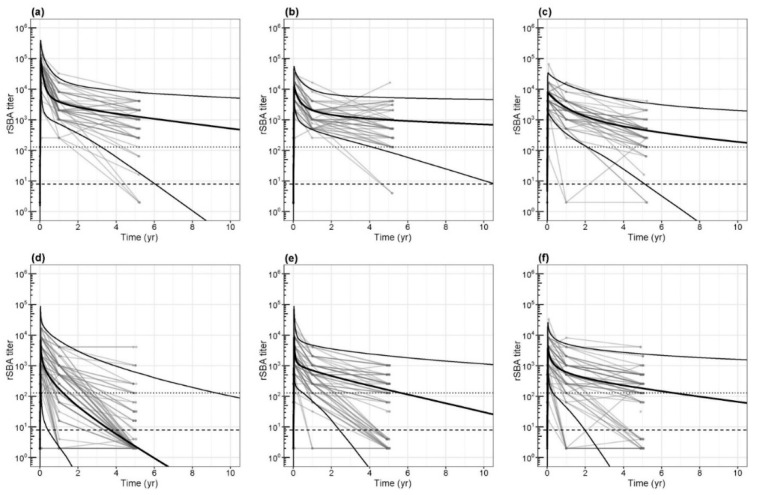
The predicted meningococcal rSBA titers for serogroup C (**a**) and (**d**), W (**b**) and (**e**) and Y (**c**) and (**f**) after a meningococcal serogroup A, C, W, Y conjugated to tetanus toxoid (MenACWY-TT) vaccination in adolescents (**a**–**c**) as a booster vaccination after being primed with a meningococcal serogroup C conjugated to tetanus toxoid (MenC-TT) vaccination at young age, and in middle-aged (**d**–**f**) adults as a primary vaccination, estimated by the bi-exponential decay model. Individual measurements are connected and presented as grey lines. Bold lines represent the 5% percentile, median, and 95% percentile rSBA titers. Dashed lines indicate correlate of protection (rSBA titer = 8) and conservative threshold of protection (rSBA titer = 128), respectively.

In adolescents, the estimated median rSBA titers of all three serogroups, using the bi-exponential decay model, remained above the correlate of protection (rSBA ≥8) for 32, 98, and 33 years for MenC, MenW, and MenY, respectively (Figure 4 and Table 2). For MenC and MenY, it was estimated that the median rSBA titer would reach the more conservative threshold of ≥128 at 17 years and 13 years postvaccination, respectively (Figure 4), while the median MenW rSBA titer was estimated to remain above this threshold for 43 years.

In contrast, the middle-aged adults showed a median rSBA titer below the threshold of 8 against MenC already after 3.7 years due to a steep decay in antibodies that continued after the first year postvaccination (Figure 4). The decay of MenW- and MenY-specific functional antibodies is less steep compared to the decay for MenC and median protection was estimated to continue up to 14 and 21 years after vaccination, respectively (Table 2).

Waning of serogroup-specific functional antibodies continues over time and follows a pattern characterized by a rapid decay in the first year and a slower decay thereafter (Table 2, Figure 4). Between one year and five years after vaccination, mean annual decay rates vary from 0.58–1.65 except for MenC antibodies in middle-aged adults that still show a mean annual decay rate of 6.65 after the first year up to the fifth year, similar to the decay rate in the first year after vaccination (10.2).

### 3.4. Correlation between Functional Antibodies and Serum IgG Concentrations

**Figure 5:**
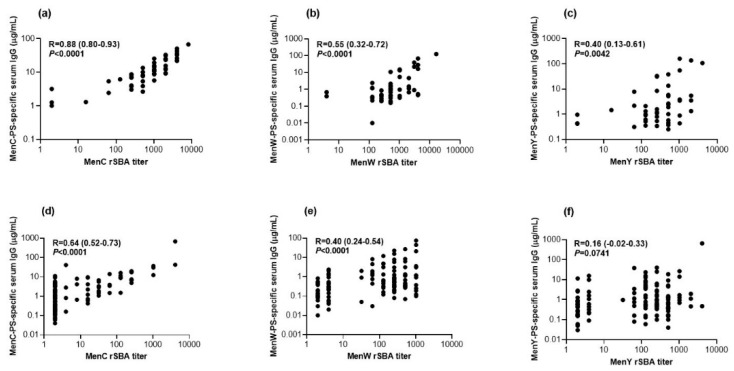
Correlation between the meningococcal serogroup C (**a**) and (**d**), W (**b**) and (**e**) and Y (**c**) and (**f**) polysaccharide (MenC, W, Y-PS) specific antibody concentrations and rSBA titers, five years after a meningococcal serogroup A, C, W, Y conjugated to tetanus toxoid (MenACWY-TT) vaccination, in both adolescents (**a**–**c**) and middle-aged adults (**d**–**f**). The correlations were analyzed using Spearman’s rho correlation test.

Comparison of the functional antibody titers using the rSBA assay with serogroup-specific serum IgG concentrations measured by MIA demonstrated a good correlation of R = 0.88 for MenC in adolescents and R = 0.64 in middle-aged adults (Figure 5).

The protected proportions for the rSBA and IgG (using the arbitrary IgG cut-off of ≥2 µg/mL) respectively are comparable for MenC in both adolescents (94% and 96%) and middle-aged adults (32% and 38%). However, 20 out of 89 (22%) of middle-aged adults with low bactericidal activity showed MenC-PS specific IgG concentrations that exceeded 2 µg/mL. A correlation of R = 0.55 was observed for MenW antibodies in adolescents, and, although adolescents showed sufficient killing against MenW in 96%, only 28% reached the MenW-PS specific IgG threshold of ≥2 µg/mL. In addition, in middle-aged adults, this discrepancy was observed, with protective titers in 65% while only 19% possessed a MenW-PS specific IgG concentration of 2 µg/mL or higher. The correlation was poor for MenY, especially in the middle-aged adults (R = 0.16).

## 4. Discussion

In this study, we estimated the long-term protection against invasive meningococcal CWY disease after a single MenACWY-TT vaccination in MenC-vaccinated adolescents and in middle-aged adults and investigated the persistence of meningococcal serum antibodies in these two age groups. Five years after a single MenACWY-TT vaccination, the proportion of adolescents with a rSBA titer ≥8 for MenCWY is 88%. Based on rSBA titers, the median duration of protection in adolescents was estimated to be 32 years against MenC, 98 years against MenW and 33 years against MenY using a bi-exponential decay model. In contrast, in middle-aged adults who received the MenACWY-TT vaccination at 50–65 years of age, only 19% possessed protective MenCWY antibody titers five years postvaccination with an estimated median duration of protection of 4, 14, and 21 years against MenC, MenW and MenY, respectively.

Five years after vaccination adolescents showed significantly higher GMTs for the meningococcal serogroups CWY compared to middle-aged adults. This difference seems due to both significantly higher peak titers one month after vaccination for all three serogroups and a lower antibody decay rate for MenC and MenW in adolescents in the years thereafter. In former studies, adolescents showed a good immune response in reaction to a MenACWY-TT vaccination [14,19,20,21,29,48,49] and protection is known to last for at least several years [50,51,52]. Information about long-term protection in middle-aged adults is, however, scarce and the available studies had a short follow-up or investigated other conjugate vaccines or the plain polysaccharide vaccine [53,54,55,56]. The only comparable study was done by Borja-Tabora et al. [26], where seroprotection in adolescents and adults 18–55 years of age was also compared five years after a MenACWY-TT vaccination. While we found significantly longer protection in adolescents for all serogroups, they found mixed differences and a remarkable significantly higher protection level against MenY in adults. These differences might be at least partly explained by the younger age of the adults (18–55) than that of the middle-aged adults of 50–65 years in our study at time of vaccination.

When naïve B cells encounter a new antigen, for instance through vaccination, the differentiation into antibody-producing B-cells and memory B cells is induced [57]. However, the response to a new antigen such as following primary vaccination might be hampered at older age, as a consequence of immunological ageing [58,59]. Antibody production is maintained either by long-lived plasma cells or a continuously active pool of memory B cells. At older age, the naïve B cell pool is limited and changes in the bone marrow affect the storage and survival of plasma cells [59]. This might explain the limited persistence of the antibody response and therefore shorter duration of protection after vaccination in the middle-aged adults compared to the adolescents, as described before [59].

The age groups in our study differed in vaccination history, with a MenC conjugate vaccination for all adolescents at young age while middle-aged adults did not receive a meningococcal vaccination earlier. This might have influenced the difference in adolescents and middle-aged adults with regard to MenC protection levels. However, van der Heiden et al. [18] observed a booster-like response for MenC in the Dutch middle-aged adult group after a first MenC conjugate vaccination. MenC-PS specific IgG concentrations increased within seven days while, for MenW and MenY, this early increase was not observed. Preexisting immunity for MenC is likely since a higher incidence of MenC disease was observed between 1998 and 2002 [12]. In the present study, it is possible that all age groups might have been exposed equally to MenC or MenW and have gained memory immunity. Adolescents in addition to natural exposure were vaccinated with MenC in early childhood. Since the mass-campaign in 2002 [60], only a very few cases of invasive meningococcal C disease occur every year in the Netherlands [61]. As a result, natural boosting for MenC after receiving the MenACWY-TT vaccination in 2014 is now very limited for our participants. We suggest that this absence of exposure might have contributed to the low MenC rSBA titers in the majority of middle-aged adults five years postvaccination and high annual decay rate of MenC IgG antibodies also in the 1–5 years postvaccination [5]. Our findings emphasize why natural boosting must be taken into account when designing vaccination strategies. Moreover, this is highlighted by our results that showed that the duration of protection in adolescents is three times longer for MenW (primary vaccination) compared to MenC (booster vaccination) and MenY (primary vaccination), possibly due to the recent MenW epidemic. Furthermore, factors related to the vaccine’s profile might play a role in the differences between serogroups. In MenACWY-TT, the MenC (and also MenA) polysaccharide is conjugated indirectly to tetanus toxoid with an adipic dihydrazide, while MenW and MenY polysaccharide are directly conjugated to the carrier [62]. It is possible that this indirect conjugation improves SBA titers just after vaccination by optimizing outward presentation of the polysaccharide on the carrier protein to immune cells [63]. The effect of conjugation process on the long-term immune response remains, to the best of our knowledge, however unknown.

Remarkably, the MenC-PS specific IgG GMC in adolescents was almost 8-fold higher than for MenW five years after vaccination, while their rSBA GMTs were comparable. Because of the circulation of MenW, other non-capsular IgG antibodies or IgM antibodies may contribute to the rSBA, while these are contributing less for MenC in the absence of recent circulation of this pathogen. As a result, the total meningococcal serum IgG concentration alone after a single vaccination might be less predictive for long-term seroprotection as defined by rSBA and, as such, the specific IgG after five years contributes only partly to the long-term protective titers. In contrast, when a strong IgG meningococcal antibody booster response is induced after revaccination or natural boosting, rSBA titers and IgG concentrations correlate better, as described earlier for a meningococcal booster vaccination [29].

The longitudinal aspect of this study with a follow-up of five years is an important strength of this study. Since the studies were performed in the same year, participants were exposed to the same natural circulation of meningococci so, when interpreting differences between age groups, timing can in that way be disregarded. Furthermore, we confirmed that median duration of protection after a meningococcal conjugate booster vaccination at adolescent age in Dutch adolescents is more than 30 years for MenC, which is in line with earlier findings by van Ravenhorst et al. [42]. Several limitations need to be considered such as the different meningococcal vaccination history in the two age groups and the large exclusion number in adolescents due to the mass campaign for MenACWY. It is worth mentioning that, within our adolescent group, three subgroups were present, vaccinated at age 10, 12, or 15 years. No clear differences in estimated duration of protection between these subgroups were observed in this study (data not shown), while, in these adolescents, significant differences were observed in serum bactericidal antibody titers between these subgroups in former studies [13,14,29]. The lack of a difference between these subgroups might be due to the low number of adolescents or to the long follow-up of five years. Furthermore, no historical rSBA data for MenA were available for the middle-aged adults and, therefore, we could not estimate long-term seroprotection for this serogroup. However, invasive MenA disease is very rare in the Netherlands [64,65] and MenA-PS specific GMCs suggested adequate seroprotection in both age groups (Figure 3).

## 5. Conclusions

In conclusion, seroprotection for MenCWY is maintained in adolescents five years after a MenACWY-TT vaccination and estimated duration of protection is more than 30 years for MenC and MenY and even lifelong for MenW with a duration of 98 years. In contrast, middle-aged adults are insufficiently protected on the long run, especially against MenC, due to faster waning of antibodies. When vaccine-induced herd protection is established, natural boosting by meningococcal circulation will be diminished or even eradicated. This must be taken into account when vaccination strategies are adapted, to protect all age groups against invasive meningococcal disease.

## Figures and Tables

**Table 1:** Meningococcal serogroup C, W, Y (MenCWY)-specific geometric mean rSBA titers (GMTs), MenACWY-polysaccharide (PS)-specific concentrations (GMCs), and proportions of participants with a serum bactericidal antibody (rSBA) titer ≥8 and ≥128 with corresponding 95% confidence intervals (CI) determined five years after vaccination. *p*-value proportions calculated with Fisher’s exact test. *p*-value difference in GMT and GMC calculated with Mann–Whitney test. *p*-value difference in proportions calculated with Wilson score interval with continuity correction.

Antibody		Age Group		*p*-Value
		Adolescents (n = 50)	Middle-Aged Adults (n = 130)	
MenA	GMC MenA-PS-specific IgG µg/mL (95% CI)	1.8 (1.2–2.5)	2.8 (2.0–3.9)	0.1532
MenC	GMT (95% CI)	588 (341–1014)	6.5 (4.6–9.0)	<0.0001
	% rSBA-titer ≥8 (95% CI)	94% (82–98)	32% (24–40)	<0.0001
	% rSBA-titer ≥128 (95% CI)	88% (75–95)	13% (8–20)	<0.0001
	GMC MenC-PS-specific IgG µg/mL (95% CI)	10.1 (7.7–13.2)	1.3 (0.9–1.7)	<0.0001
MenW	GMT (95% CI)	578 (372–898)	52.5 (35.4–78.3)	<0.0001
	% rSBA-titer ≥8 (95% CI)	96% (85–99)	65% (56–73)	<0.0001
	% rSBA-titer ≥128 (95% CI)	96% (85–99)	56% (47–65)	<0.0001
	GMC MenW-PS-specific IgG µg/mL (95% CI)	1.3 (0.8–2.1)	0.5 (0.4–0.6)	0.0008
MenY	GMT (95% CI)	270 (170–430)	70.5 (47.2–105)	0.0002
	% rSBA-titer ≥8 (95% CI)	94% (82–98)	71% (62–79)	0.0006
	% rSBA-titer ≥128 (95% CI)	86% (73–94)	64% (55–72)	0.0036
	GMC MenY-PS-specific IgG µg/mL (95% CI)	2.2 (1.3–3.6)	0.9 (0.7–1.2)	0.0045

**Table 2:** Fold changes and mean annual decay rates (relative decrease) in meningococcal serogroup C, W, Y (MenCWY)-specific geometric mean titers, and minimal (2.5% percentile) and median duration of protection (median rSBA titer ≥8).

Antibody		Adolescents	Middle-Aged Adults
MenC	Fold-change 1 month vs. 1 year	14.3	10.2
	Fold-change 1 year vs. 5 years	4.7	26.6
	Mean annual decay rate 1–5 years	1.18	6.65
	Minimal duration of protection	4.6 years	0.2 years
	Median duration of protection	32.4 years	3.7 years
MenW	Fold-change 1 month vs. 1 year	5.1	5.3
	Fold-change 1 year vs. 5 years	2.3	6.6
	Mean annual decay rate 1–5 years	0.58	1.65
	Minimal duration of protection	7.0 years	1.7 years
	Median duration of protection	97.7 years	13.9 years
MenY	Fold-change 1 month vs. 1 year	3.2	6.1
	Fold-change 1 year vs. 5 years	5.4	4.2
	Mean annual decay rate 1–5 years	1.35	1.05
	Minimal duration of protection	3.7 years	1.4 years
	Median duration of protection	33.4 years	20.8 years
